# Diabetes mellitus as etiological factor of hearing loss

**DOI:** 10.1016/S1808-8694(15)31312-4

**Published:** 2015-10-20

**Authors:** Clícia Adriana S. Maia, Carlos Alberto H. de Campos

**Affiliations:** ^1^ Otorhinolaryngologist; Supporting Professor, Medical School, Universidade Estadual Santa Cruz; Post-graduation in Otorhinolaryngology under course, Faculdade de Ciências Médicas da Santa Casa de São Paulo; ^2^ Joint Professor, Head of the Department of Otorhinolaryngology, Faculdade de Ciências Médicas da Santa Casa de São Paulo

**Keywords:** hearing loss, diabetic angiopathy, diabetes mellitus, retinopathy, neuropathy

## Abstract

Patients with *diabetes mellitus* often show symptoms such as dizziness, tinnitus, and hearing impairment. In general, hearing loss is sensorineural, which is sometimes confused with presbycusis, mainly because it develops in patients older than 40 years of age. Angiopathy and neuropathy caused by diabetes mellitus have been considered important factors for the vestibular-cochlear disorders found in these patients. However, there is controversy regarding the etiopathogenesis of hearing loss, as some researchers support that it develops due to neuropathy, others say it is due to angiopathy, or even a combination of both. Yet, some researchers believe diabetes mellitus and hearing loss are part of a genetic syndrome. We have conducted an extensive bibliographic review to determine whether there is cause-effect relationship between diabetes mellitus and hearing loss. We were able to verify that, despite the large number of studies performed, there is still a great deal of controversy, and new approaches are being studied, for example in the field of genetics, which shows that new paths can be followed to reach a conclusion on this issue.

## INTRODUCTION

The most frequent causes of vestibular and auditory abnormalities are attributed to dysfunctions in the metabolism of carbohydrates, thyroid affections, supra adrenal, and other different metabolic disorders. Among glucose metabolism disorders, diabetes mellitus is the affection most commonly related with auditory disorders^1^.

Diabetes mellitus is a genetically determined metabolic disorder associated with absolute or relative impairment of insulin and in complete clinical manifestation is characterized by metabolic affections, vascular and neuropathic complications. The main objective of treating patients with diabetes is the prevention of chronic complications because the disease is not curable but only controllable. In Brazil, the incidence of chronic complications of diabetes is quite high. It is estimated that there are five million subjects with diabetes and half of them are not aware of the diagnosis. A large number of subjects, especially children and adolescents, have diagnosis of diabetes made in face of complications, especially infections^2^.

One of the morphological aspects more constant in diabetes mellitus is diffuse thickness of basal membrane, which may also happen with vascular endothelium, and it is named diabetic microangiopathy. It is more evident in skin capillaries, skeletal muscles, retina, renal glomeruli, and renal medulla. Its pathogenesis is still obscure, but it is clearly associated with hyperglycemia. Other morphological affections refer to impairment of both motor and sensorial nerves of lower limbs, characterized as Schwann cell lesions, degeneration of myelin and axon damage. The cause of neuropathy is still very controversial, and it may be related with diffuse microangiopathy that would affect nourishment of peripheral nerves^3^.

Neuropathy and angiopathy are common affections in diabetes mellitus. Angiopathy has been observed in small arteries and skin capillaries, muscle, kidney, retina and peripheral nerves. The factors that may cause neuropathy are metabolic disorders (glucose metabolism, lipid metabolism defects and vitamins). Some researchers referred to the fact that vascular affections in interfascicular or intrafascicular branches of vasonbervorum contribute to neuropathy. Atherosclerosis, however, very common in diabetes mellitus, can also contribute to neuropathy, owing to interference in rate of nutrient transfer^4^.

Angiopathy may occur both in direct way, interfering with supply to the cochlea by reducing transport through the thickened walls of capillaries, and indirectly by the reduction of flow in vascular pathways, or still, because of secondary degeneration of 8th cranial nerve^5^.

Concerning diabetes mellitus, there are different opinions about the pathological affections caused in the auditory system.

According to Nageris et al., the association between hearing loss and diabetes mellitus has been debated since it was first reported by Jordao in 1857^6^.

In the literature, there are many different types of hearing loss found in diabetic patients. One of them is progressive, gradual bilateral sensorineural loss, affecting especially high frequencies and the elderly. It would be similar to presbyacusis, but with more severe losses than those expected by aging^5^, ^6^, ^7^, ^8^. Conversely, there are authors that report the possibility of having early sensorineural loss^7^ and others that reported hearing loss in low and medium frequencies^9^, ^10^. Some studies described diabetes as the possible cause of unilateral sudden loss^4^, ^10^, ^11^, but other authors did not find the same association^12^.

As to incidence of hearing loss in patients with diabetes mellitus, we observed that there is no consensus in the literature either, ranging from zero to 93%^6^.

In view of the reported data, we decided to perform a literature review to check whether there is really a hearing loss inherit to diabetes mellitus and to which factors it is related.

## LITERATURE REVIEW

### A) Diabetic angiopathy: structural and ultrastructural aspects

Diabetic angiopathy has been characterized by endothelial proliferation, accumulation of glucoprotein in the intima and thickness of basal membrane of capillaries and small blood vessels. We can also observe fibrotic thickness of the wall and narrowing of internal auditory artery lumen. We confirmed accumulation of positive PAS substance (periodical Schiff acid) on the artery wall, as well as on the modiolus vessels and vascular stria capillaries^4^.

#### A1) Animal lab experimental studies

The effects of diabetes in the inner ear can be studied in experimental model by using diabetogenic drugs (alloxan or streptozocin), in genetically modified animals with diabetes (such as Sabra line rats) by means of total or subtotal pancreatectomy and still by extraction of anterior hypophysis.

Costa^13^ (1967) used alloxan to induce diabetes in rats to assess diabetic angiopathy. They found optical microscopy with increase in thickness of vessel walls of modiolus, more marked six months after the induction of diabetes. We did not observe structural changes to Corti's organ, in ganglionar cells or in nervous fibers.

Smith et al.^14^ (1995) confirmed that diabetic microangiopathy occurred in the inner ear of rats induced with the use of streptozocin. In the studied group, there was quantitative documentation by electron transmission microscopy of the increase in thickness of capillary basal membrane of vascular stria, which was not observed in the control group.

Raynor et al.^15^ (1995) also used streptozocin to induce diabetes mellitus and observed that loss of outer hair cells was greater in diabetic rats that were simultaneously exposed to noise.

The use of alloxan or intraperitoneal streptozocin to induce diabetes in experimental models has been contested because it did not reflect the real physiological mechanism of diabetes in human beings because it has genetic origin. Thus, Nageris et al.^6^ (1998) used rats of Sabra line who have the tendency to presenting intolerance to glucose when submitted to 21 days of rich carbohydrate diet. They concluded that if there is sensorineural loss associated with diabetes mellitus, its pathogenesis does not involve damage to hair cells or stria vascularis.

Triana et al.^16^ (1991), using rat line (SHR/N-cp – Spontaneous hypertensive rats/corpulent), who stated that this is the only genetic model for non-insulin-dependent diabetes mellitus, observed significant loss of outer hair cells in animals that were simultaneously obese and diabetic, when compared to the control group. However, a similar experiment was conducted with other authors using the same animal mode and they concluded that diabetes alone did not cause significant thickness of basal membrane, but in combination with obesity and exposure to noise they presented thickness, which was considered significant^17^.

#### A2) Anatomic pathological study in humans

Willianson & Killo^18^ (1977), in a review study mentioned that basal membrane of the capillary is formed by collagen proteins that are common in the connective tissue and also in scarring tissues, given that synthesis of these substances increase in response to a variety of stimuli and damage. They stated that thickness of basal membrane of the capillary associated with diabetes is considered a proliferative response of capillary cells to damage. They reported that this thickness is attributed to repetitive episodes of death of endothelial cells and their regeneration (with retention of necrotic endothelial cells on the basal membrane and beginning of new layers of the same membrane by regeneration). They concluded that a great variety of in vivo and in vitro studies are also consistent with this hypothesis, suggesting that synthesis of basal membrane and tissue renovation are increased in diabetes and that degradation of basal membrane is reduced or damaged.

Jorgensen & Buch^9^ (1961), in a study with temporal bones of 32 diabetic subjects of all age ranges, showed that the main affections are shown in preparations of stria vascularis blood vessels when stained by PAS periodical Schiff acid – a staining method that clearly defines the basal membrane, mesangial matrix and hyalinosis. In this study, we found massive deposits of PAS on the capillary walls of stria vascularis, which were 10 to 20 times thicker than the usual, and had homogeneous or lamellar structure. Abnormalities were similar to those seen in atherosclerosis, but more marked, occurring only in the stria vascularis. These authors observed degeneration in other parts of the labyrinth, but they corresponded to findings in temporal bones of non-diabetic people of the same age range. The study confirmed that angiopathy is not generalized, and it is preferably directed to some capillary systems of inner ear, such as for example, stria vascularis. However, based on these investigation, the authors state that they can not come to conclusions about the chemical nature of the precipitate, because many substances are stained by this method. They reported that affections are typical to diabetes and not specific for the disease, observing that atherosclerosis is peripherally reduced, whereas diabetic angiopathy increases its intensity in the region of small vessels.

Costa^13^ (1967), in a study conducted with temporal bone of six subjects that were used in their comparative study previously reported, have also detected positive PAS material on the wall of stria vascularis capillaries and modiolus, in addition to thickness of basal membrane in both sites.

Makishima and Tanaka^4^ (1971) used the temporal bone of four subjects and also found impregnation by periodical Schiff acid on thickness of capitally walls of stria vascularis and modiolus, which was considered responsible for narrowing of vessel lumen, more intensively affected by inner auditory artery.

After these initial data on diabetes mellitus and hearing, most researchers agreed that primary lesions are angiopathic^4^, ^10^, ^14^

### B) Diabetic neuropathy: structural and audiological aspects

#### B1) Clinical pathology studies in humans

Makishima & Tanaka^4^ (1971) described atrophy of spiral ganglion neurons and demyelization of the 8th cranial nerve in four diabetic subjects. They showed demyelization is also the initial lesion of peripheral nerves of diabetes extremity and that there are indications of abnormalities of myelin metabolism that may have significant importance in pathogenesis of diabetic neuropathy. Using optical microscopy, we observed demyelization of auditory nerve by defferentation of myelin sheath with small affections to axon and fibrosis of perineurum, severe atrophy of spiral ganglion with loss of cell of basal turn and medium turn of the cochlea, in addition to decrease in number of nervous fibers on the spiral lamina. Other findings were: reduction of number of ganglionar cells of ventral and dorsal cochlear nuclei, small loss of ganglionar cells of superior olivary nucleus, inferior colliculus and medial geniculate body. In auditory centers of both temporal bone, they did not detect any specific affections directly attributed to diabetes mellitus.

#### B2) Audiological study in human

There are other authors that believe that neuropathy is the primary lesion of hearing loss, arguing that positive PAS material found in the vessel wall is very nonspecific, found also in other diseases. They conducted an audiometric study in 20 patients with diabetic peripheral neuropathy using the control of 32 patients with diabetes mellitus. They observed that the thresholds of peripheral neuropathy subjects were also worse than the control group, in all frequencies. And also that patients aged over 60 years were worse than both groups. Hearing loss found by them was normally sensorineural, progressive and more intense when over the age of 60 years^7^.

### C) Correlation between angiopathy and/or diabetic neuropathy and hearing loss

Table 1 presented the studies that referred to finding a relation between diabetes mellitus and hearing loss and Table 2 presents studies that did not find a correlation between them.

**Table 1:** List of studies that found correlation between diabetes mellitus and hearing loss.

Author	Number of patients	Gender Influence of gender	Age Influence of age	Type of DM Influence of duration	Relation w/presence of complications DM	Audiological results	Control group
Camisasca et al. (1950)	81	male > fem	29-75; absent	I; present	present	DSN in 46% of the cases	no data
Jorgensen & Buch (1961)	69	52♂G17♀; absent	16-73; present	I; absent	w/retinopathy and nephropathy	41% DSN bilateral	Johansen curve (1943)
Tota & Bocci (1965)	100	39B♂61♀; no data	11-80; present	I and II; present	retinopathy	9dB – 6KHz; 15dB – 3KHz, > 61-70 years	Patients w/out DM matched by gender
Marulo et al. (1974)	60	36B♂24♀; no data	20-49; present	I and II; present	retinopathy, coronaryopathy peripheral angiopathy	DSN in 30% of the cases	Johansen curve (1943)
Friedman et al. (1975)	20	8B♂12♀; no data	22-70; present	II; present	peripheral neuropathy retinopathy and use of AB	DNS in 55% of the cases	32 patients matched by age
Taylor & Irwin (1978)	77	17B♂21♀; fem > male	15-62; present	I; absent	absent	DSN mild below 9dB.	39 patients
Ferrer et al. (1991)	46	no data	14-40; present	I; present	retinopathy and nephropathy	30dB in at least one frequency	matches by age
Cullen & Cinamond (1993)	44	male > fem	mean: 46.9; present	I; absent	no data	p < 0.001 high freq.	38 matched by age and gender
Tay et al. (1995)	102	58B♂44♀; no data	19-80; absent	59 – I 43- II/present	no correlation	low and medium frequencies p < 0.001	matched by gender
Dalton et al. (1998)	344	no data	43-84; present	II; absent	No association w/retinopathy. Association w/nephropathy	high frequencies above 4000Hz	absent
Karkalapudi et al. (2003)	12575	no association	No association	No data	Poor levels of creatinine and microvascular disease	13.1% prevalence	53461 non diabetic

**DM** – Diabetes mellitus; **dB** – decibels; **DSN** – sensorineural hearing loss; **AB** – antibiotics; **Johansen Curve (1943)** – results of audiometric exams found by the author in subjects of different age ranges in a specific population.

**Table 2:** Studies that do not find correlation between diabetes mellitus and hearing loss.

Author	Number of patients	Gender Influence of gender	Age Influence of age	Type of DM Influence of duration	Relation w/presence of complications DM	Affected frequencies loss in dB	Control group
Profazio & Barraveli (1959)	40	no data	9-70; present	I; present	Neuropathy retinopathy and use of AB	DSN in 55% of the cases > 44 years	Johansen curve (1943)
Strauss et al. (1982)	660	I-59% ♂	I-mean: 35:	I e II; absent	Other factors: hypertension and noise exposure	significant abnormalities were not found	non-diabetic population
		II-30% ♂					
		III-35% ♂ no data	II e III-mean: 65; present				
Miller et al. (1983)	33	18B&/15♀; no data	22-72; present	I; absent	Other factors: noise exposure	significant abnormalities were not found	209
Axellson & Fagerberg (1968)	99	59B&/40♀; absent	16-59; present	I; absent	no correlation	significant abnormalities were not found	Non-diabetic population
España et al. (1995)	47	I-8B&/9♀	7-47; present	p = 0.0143	no correlation	DSN in 30% of the cases	30 healthy patients
		II-10B&/20♀; no data					

**DM** – Diabetes mellitus; **dB** – decibels; **DSN** – sensorineural hearing loss; **AB** – antibiotics; **Johansen Curve (1943)** – results of audiometric exams found by the author in subjects of different age ranges in a specific population.

### D) Correlation between genetic affection, diabetes mellitus and hearing loss

It is known that hearing loss can also have its origin from genetic mutations, which can be congenital or acquired, occur in nuclear genes and mitochondria and present syndromic or non-syndromic episodes. DNA mitochondrial mutations are transmitted by maternal line, but there may be spontaneous mutations. As to physiopathogenesis of hearing loss, there are still many issues and theories^19^. Yamasoba et al^20^ (1996) presented the following theory: mitochondria 1 DNA affection → alteration in microchondril protein synthesis → alteration in oxiphative phosphorilation → reduction in the formation of ATP → alteration of ionic pumps → alterations in potassium, sodium and calcium → cell death.

In 1989, a specific genealogical tree was studied by Lemkes et al.^21^, in which nine children with diabetes mellitus also showed hearing loss.

Authors have observed a coinciding loss of variable severity in each diabetic patients. When the third-generation line was considered, they detected diabetes and hearing loss present only in children of affected mothers. This observation has strongly suggested that heritage is exclusively maternal, which is a particular characteristics of diseases associated with mitochondrial DNA mutation.

Newkirk et al.^22^ (1997) reported in a prospective study prevalence of diabetes mellitus and maternal inherited deafness in the population of diabetic patients at Newcastle Hospital. They reported that this association was a new subtype of diabetes and that it resulted from substitution of adenine for guanine at position 3243 of mitochondrial gene tRNA^Leu(uur)^. They also pointed out that this syndrome was originally identified as a cause of MELAS syndrome (myopathy, encephalopathy, lactic acidosis and repetitive episodes of cerebral vascular accident) and that sensorineural hearing loss was an additional symptom in about 70% of the cases.

In some cases, the same mutation may cause different phenotypes such as in the case of gene tRNA^leu^, a substitution of nucleotide adenine for guanine in locus 3243, which may cause three different phenotypes: progressive chronic external ophthalmoplegia, diabetes, maternal-originated deafness and MELAS syndrome. Clinically, diabetes mellitus and maternal inherited deafness are characterized by insulin-dependent diabetes, which can be non-insulin dependent at first, but normally evolves to it because the mitochondrial affection modifies insulin secretion by the pancreas; patients are thin and less than 40 years of age and hearing loss is normally sensorineural progressive^19^.

Fowler et al.^23^ (1999) described Diadmoad syndrome or Wolfram syndrome also as a consequent mutation of mitochondrial DNA, characterized by diabetes insipidus and mellitus, optical atrophy and sensorineural hearing loss starting at childhood.

## DISCUSSION

As presented in the literature review, there is not enough evidence to solidly define diabetes mellitus as a cause of hearing loss. There is no consensus on any aspect of this topic, either audiologically or histopathologically. Clinical experience, in many studies, showed direct correlation between hearing loss and diabetes, as reported in Table 1. However, there are other studies with more subjects and better designed that did not identify this association (Table 2).

Affection of blood vessels that irrigate the inner ear and affections that occur in stria vascularis of diabetic patients are unquestionable facts confirmed by different authors that believe in the correlation between hearing loss and diabetes, and these affections are strong evidences that diabetes may cause hearing loss^10^, ^13^, ^14^, ^15^. However, there are others that do not believe in this association^24^.

Some studies indicate that decrease in hearing acuity, similarly to the one presented in presbyacusis, is greater than what is expected for the age in elderly and diabetic patients^8^, ^10^, ^12^, ^22^, ^24^. According to them, it is more likely that we can associated factors that would lead to hearing loss, such as consequence of vascular abnormalities caused by diabetes mellitus.

Up to 1960, there were 3 theories about the pathogenesis of hearing loss caused by diabetes mellitus: neuropathy, angiopathy and association of both. Currently, there are significant reasons to believe that the angiopathy found in diabetic patients contributes to greater evidences of hearing loss. In favor of this hypothesis we can rely on histopathological findings of microvascular lesions of the inner ear, such as thickness of basal membrane of stria vascularis capillaries, as well as the fact that diffuse vascular affections caused by diabetes mellitus are well known^4^, ^13^, ^14^, ^15^, ^25^, ^26^.

It is known that the main difficulty to conduct inner ear study is related with difficulty to access histological assessment, especially in human beings. Moreover, the steps to preparation of material can also have artifacts, provoking misinterpretations. The localization of inner ear circulation makes it difficult to study homeostasis, as well as the consequences of metabolic disorders. Other factors that can induce misinterpretation of statistical data are use of different populations for comparison purposes, lack of good control group, need to conduct longitudinal studies (owing to the fact that in transversal studies many times it is not possible to know if diabetes mellitus precedes hearing loss or vice-versa). Other important aspect is size of studied group and the huge universe of variables such as other diseases, use of drugs, noise exposure, genetic affections. It is possible that these are the reasons for discrepancy in results detected among similar studies conducted by different authors.

Finally, we observed growing interest in hearing loss caused by genetic affections, many of them related to mitochondrial DNA mutations. They correspond to 0.5% to 1% of all genetically-based hearing losses, which may be associated with syndromic presentations. One of the syndrome presentations is diabetes mellitus associated with maternal-inherited hearing loss, amounting to 1.5% of all cases of diabetes in Japan and The Netherlands^19^.

The investigation of genetic causes has been the answer to many questions and disagreeing results.

## CLOSING REMARKS

In view of current knowledge, there is evidence that diabetes mellitus may cause hearing loss, but we can not state that there is clear cause-effect correlation. It is known that a series of variants may favor the association between both diseases, but it is necessary to conduct more careful studies to define the true role of these factors. As seen before, diabetes mellitus and hearing loss can be dependent components, or even components of a genetic syndrome and not dependent one on the other.

According to the analyzed studies, we can conclude that many questions will be answered by multicenter longitudinal studies with large populations and strict inclusion and exclusion criteria.
